# Serum Tenascin-C and Alarin Levels Are Associated with Cardiovascular Diseases in Type 2 Diabetes Mellitus

**DOI:** 10.1155/2022/2009724

**Published:** 2022-04-21

**Authors:** Mingming Li, Mengjiao Wu, Hua Zhu, Yulin Hua, Zijun Ma, Jiayi Yao, Bin Feng, Bimin Shi

**Affiliations:** Department of Endocrinology and Metabolism, The First Affiliated Hospital of Soochow University, Suzhou 215006, China

## Abstract

**Background:**

Tenascin-C (TNC), an extracellular matrix glycoprotein, is elevated in inflammatory and cardiovascular pathologies, whereas alarin, a novel orexigenic peptide, participates in insulin resistance and glycometabolism. The roles of these molecules in individuals with cardiovascular disease (CVD) and type 2 diabetes mellitus (T2DM), clinical conditions associating with metabolic disorders, and chronic inflammation, remain controversial. Our study aimed at determining the potential role of TNC and alarin in CVD adult patients with T2DM.

**Methods:**

This was a cross-sectional study. Basic and clinical information for 250 patients with T2DM were analyzed. Based on their cardiovascular disease status, participants were assigned into the CVD and non-CVD groups. Serum TNC and alarin levels were assessed by enzyme-linked immunosorbent assay (ELISA).

**Results:**

Serum TNC and alarin concentrations in the CVD group were significantly higher than those of the non-CVD group. Moreover, serum TNC levels were positively correlated with age, waist circumference, and waist-hip ratio; however, they were negatively correlated with TC, LDL-C, and eGFR levels. Alarin levels were positively correlated with BMI, waist circumference, and hip circumference. In logistic regression models, TNC and alarin were also established to be independent determinants for CVD in T2DM patients and their increases were associated with CVD severity. Receiver operating characteristic (ROC) curve analysis showed that the area under curve (AUC) values for TNC and alarin were 0.68 and 0.67, respectively. TNC and alarin were good predictors of CVD occurrence. When the cutoff value for TNC was 134.05 pg/mL, its sensitivity was 69.47% while its specificity was 61.29%. When the cutoff value for alarin was 142.69 pg/mL, sensitivity and specificity were 38.95% and 90.97%, respectively.

**Conclusion:**

Elevated TNC and alarin levels are independently associated with the occurrence and severity of CVD in T2DM individuals. Therefore, these two biomarkers are potential diagnostic and prognostic indicators for CVD in diabetics.

## 1. Introduction

Diabetes, a metabolic disorder that is characterized by hyperglycemia, develops as a result of insulin resistance, a relative lack of insulin, or both. Currently, about 130 million Chinese adults are suffering from diabetes, and its prevalence is rapidly increasing [1]. Type 2 diabetes mellitus (T2DM) is relatively common, accounting for more than 90% of all cases. Cardiovascular diseases (CVDs) are a group of disorders of blood vessels. These disorders consist of coronary heart diseases (CADs), cerebrovascular diseases, and peripheral arterial diseases. Diabetes is a major risk factor for CVD, and in turn, CVD is the most common cause of morbidity, mortality, and healthcare costs in diabetic patients [2]. Compared to healthy individuals, patients with T2DM have a 3–4 fold risk for CVD-related mortality [3]. Therefore, early diagnosis and prompt intervention of CVD in T2DM patients is essential to reduce the risk of CVD-related mortalities and improve prognostic outcomes. Identification of new predictive biomarkers for CVD in T2DM patients remains imperative.

Tenascin-C (TNC), a large hexametric extracellular matrix (ECM) glycoprotein, is transiently expressed during embryonic development [4]. Although TNC is almost not expressed in most fully developed organs, it is significantly upregulated at various sites of pathological conditions, such as tissue injury and inflammation [5]. In the existing literature, TNC contributes to inflammation and atherosclerosis progression in diabetic patients. TNC is a member of ECM, and ECM remodeling has been shown to occur in diabetes and insulin resistance models [6]. Elevated levels of this protein have been reported in the retinas of diabetic patients and in chronic kidney disease patients [7, 8]. Elevated TNC levels are associated with atherosclerosis and increasing severity of atherosclerotic plaque instability [9]. But in patients with dysglycemia, these conclusions are incompatible. A cohort study in Italy found that TNC was an only independent determinant of death but unrelated with nonfatal myocardial infarction, heart failure hospitalization or revascularization and nonfatal stroke [10]. In another French prospective cohort, increased serum TNC concentrations were independently associated with nonfatal myocardial infarction, nonfatal stroke and death [11]. Various biomarkers that regulate food intake and energy expenditure play vital roles in atherosclerosis progression by regulating endothelial and chronic inflammation in diabetes patients [12, 13]. Alarin, the third member of the galanin neuropeptide family, is a neuromediator of orexigenic activities and energy homeostasis [14]. It is composed of 25 amino acids and originates as a splice variant of the galanin-like peptide gene [15]. Intracerebroventricular injection of alarin in rats stimulated food intake and increased their weights [16, 17]. Besides, elevated levels of circulating alarin have been reported in diabetes and metabolic syndrome patients [18, 19]. Alarin is associated with coronary heart disease. However, a limited number of studies have investigated the potential of serum alarin as a risk factor, or as a diagnostic marker for CVD in T2DM patients. Therefore, identifying the risk factors of CVD in T2DM patients is greatly important for early recognition and intervention of vascular events. The different serum TNC and alarin concentrations could be able to indicate the different progression of long-term complications, which have instructing functions to the secondary and tertiary prevention of diabetes.

This study aimed at investigating the significance of serum TNC and alarin levels on the presence and severity of CVDs in T2DM patients.

## 2. Methods

### 2.1. Study Population and Design

This was a cross-sectional study involving 250 patients with T2DM admitted at the First Affiliated Hospital of Soochow University, Suzhou, China, from June 1, 2020 to June 30, 2021. The inclusion criteria were as follows: Patients with T2DM according to the American Diabetes Association (ADA) criteria and aged over eighteen years [20]. The exclusion criteria were patients with: (i) other types of diabetes; (ii) severe liver dysfunction, acute infection, active neoplasia, pregnancy, and lactation; and (iii) mental disorders or autoimmune disease. We used the PASS 15.0 software to calculate the sample size. A 95% confidence and two-sided intervals were adopted. The effective sample size in each group was *N* = 57, considering a 10 percent non-response rate. Study protocols were approved by Medical Ethics Committee of the First Affiliated Hospital of Soochow University, and all participants provided written informed consents.

At enrollment, clinical histories of the participants were investigated by well-trained and experienced physicians. Participants were classified as having had CVD events if a history of CAD, cerebrovascular diseases, or peripheral arterial diseases (PAD) was evident. The definition of CAD consists of a history of hospitalization for angina pectoris, myocardial infarction, and ischemic heart disease, or a surgical history of coronary stent implantation, coronary balloon angioplasty or coronary arterial bypass. Cerebrovascular diseases were confirmed as a history of hemorrhagic or ischemic stroke, diagnosed by imaging detections (computed tomography or magnetic resonance imaging). PAD was defined as an ankle-brachial index <0.90 in either leg. In addition, patients exhibiting the presence of intermittent claudication, resting pain, non-healing distal ulcers, or gangrene as well as a history of lower limb percutaneous transluminal angioplasty were considered to have PAD. CVD was also diagnosed by reviewing the medical records for all study participants, and physical examination, electrocardiogram, carotid ultrasonography were performed on all participants. Identification of various non-CVDs were based on self-reports of patients, and confirmed by their medical records.

### 2.2. Anthropometric and Biochemical Measurements

We used a standard questionnaire to collect personal data. Variables were recorded as follows: demographic characteristics (age and gender), smoking habits, duration of diabetes, past history of hypertension or dyslipidemia, and previous medication. Smoking was defined as smoking more than 1 cigarette per day for more than 6 months. Hypertension was diagnosed when systolic blood pressure ≥140 mmHg, diastolic blood pressure ≥90 mmHg (at least two times in different environments) or if administered with antihypertensive drugs [21]. Dyslipidemia was defined as triglyceride levels >150 mg/dL, high-density lipoprotein cholesterol <40 mg/dL, or indications to hypocholesterolemic medications [22]. Diagnosis of diabetic nephropathy (DN) and diabetic retinopathy (DR) were based on the ADA criteria [20].

After anthropometric measurements, body weight (accuracy 1 kg), height, (accuracy 1 cm), and hip and waist circumference (accuracy 1 cm) were recorded. Body mass index (BMI) was calculated as weight (kg)/height squared (m^2^). Waist-hip ratio (WHR) was defined as the ratio of waist circumference to hip circumference. Carotid ultrasonography was performed by experienced sonographers using high-resolution and linear-array transducers (IU22, Philips, Netherlands). A Doppler probe was used to obtain the ankle-brachial index (ABI), which is the ratio of the highest systolic pressure measured in each leg to the higher of the left or right arm brachial artery pressure.

All participants were asked to fast for at least 12 h. Peripheral blood samples were obtained at 0 and 120 min following consumption of a steamed bun containing approximately 75 g glucose. Blood samples were cold-chained and transported to the laboratory for assessments within 4–6 h. The remaining samples were frozen at −80°C. Fasting blood glucose (FBG), total cholesterol (TC), triglycerides (TG), high-density lipoprotein cholesterol (HDL-C), low-density lipoprotein cholesterol (LDL-C), blood creatinine (Cr) and blood uric acid (UA) were tested using an automatic biochemistry analyzer (HITACHI 7600, Japan) through standard enzymatic methods. Glycated hemoglobin (HbA1c) was assessed by high-performance liquid chromatography (HLC-723G8, TOSOH Company, Japan). Fasting insulin (FINS), fasting C-peptide (FCP), 2 h postprandial insulin (2hINS), and 2 h postprandial C-peptide (2hCP) were determined by electrochemical luminescence immunoassay (AIA-2000ST, TOSOH Company, Japan). Estimated glomerular filtration rate (eGFR) was evaluated on the basis of the chronic kidney disease epidemiology collaboration (CKD-EPI) equation. Insulin resistance status was calculated by homeostasis model assessment of insulin resistance index (HOMA-IR). HOMA-IR = (FBG (mmol/L) × Fasting insulin (mIU/L))/22.5. Serum TNC and alarin levels were assessed using a commercially available enzyme-linked immunosorbent assay (ELISA) kit (Kanglang Biological, China) following the manufacturer's instructions.

### 2.3. Statistical Analysis

Statistical analyses were conducted using the SPSS 25.0 software, while the GraphPad Prism 8.0 software was used to draw graphs. The Shapiro–Wilk test was performed to confirm data distribution patterns. Continuous variables were expressed as mean ± standard deviation (SD) (for normally distributed data) or median (interquartile range) (for abnormally distributed data). Variables were compared by a *t*-test (for normally distributed data) or Mann–Whitney *U* test (for abnormally distributed data). Categorical variables are presented as frequencies (percentages) and were compared by a chi-square test. Variables with *p* < 0.05 in univariate analysis were incorporated into binary logistic regression models to determine independent factors for the prevalence of CVD in T2DM. Correlations between TNC or alarin and other clinical indicators were assessed by Spearman's rho test. Area under the receiver operating characteristic (ROC) curve was used to assess the discriminative ability of tenascin-C or alarin to determine CVD. *p* ≤ 0.05 was considered to be statistically significant.

## 3. Results

Among the 250 participants with T2DM, 95 had CVD, of which 35 (36.84%) had single coronary heart diseases, 52 (54.74%) had single cerebrovascular diseases, 25 (26.32%) had single peripheral arterial diseases, and 21 (22.11%) had developed at least two types of CVD. Sociodemographic clinical characteristics of the study participants are shown in Table 1. Patients with a history of CVD were elderly and they exhibited: bigger waist circumferences, longer duration of diabetes, higher proportions of hypertension and diabetic nephropathy as well as lower HbAlc, TC, LDL-C and eGFR levels. There were no significant differences between the two groups with regards to sex, BMI, hip circumference, WHR, smoking status, FBG, FCP, 2hCP, HOMA-IR, TG, HDL-C, UA, and prevalence of diabetic retinopathy. Median TNC and alarin levels were significantly higher in the CVD group, relative to the non-CVD group (*p* < 0.001). Insulin injections were common in CVD patients. Meanwhile, the CVD group was highly associated with the use of statins, renin–angiotensin–aldosterone system (RAAS) inhibitors and diuretics (Table 2).

**Figure 1:**
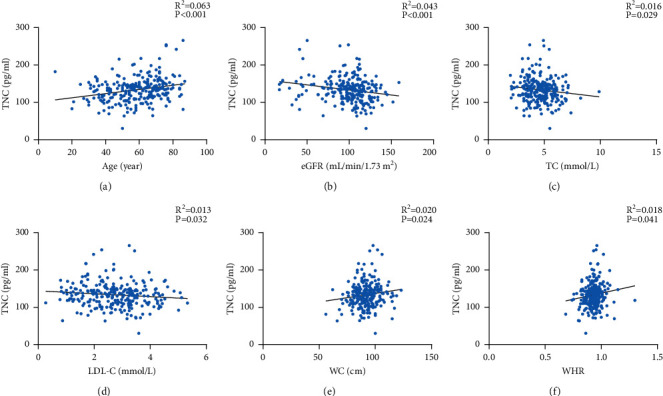
Correlation between TNC and the other clinical indicators. (a) Positive correlation between TNC and age (*r* = 0.241, *p* < 0.001). (b) Negative correlation between TNC and eGFR (*r* = −0.022, *p* < 0.001). (c) Negative correlation between TNC and TC (*r* = −0.138, *p*=0.029). (d) Negative correlation between TNC and LDL-C (*r* = −0.136, *p*=0.032). (e) Positive correlation between TNC and WC (*r* = 0.142, *p*=0.024). (f) Positive correlation between TNC and WHR (*r* = 0.129, *p*=0.041). eGFR, estimated glomerular filtration rate; LDL-C, low-density lipoprotein cholesterol; TC, total cholesterol; TNC, tenascin-C; WC, waist circumference; WHR, waist-hip ratio.

**Figure 2:**
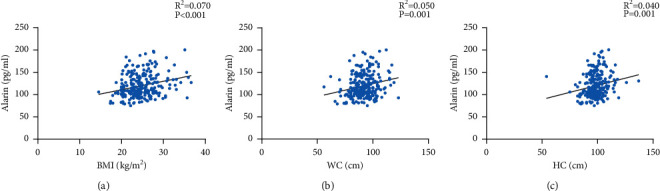
Correlation between alarin and the other clinical indicators. (a) Positive correlation between alarin and BMI (*r* = 0.253, *p* < 0.001). (b) Positive correlation between alarin and waist circumference (*r* = 0.241, *p*=0.001). (c) Positive correlation between alarin and hip circumference (*r* = 0.216, *p*=0.001). BMI, body mass index; HC, hip circumference; WC, waist circumference.

Correlations between TNC or alarin and other clinical indicators are shown in Figures 1 and 2. As TNC levels increased, so did age (*r* = 0.241, *p* < 0.01), waist circumference (*r* = 0.142, *p*=0.02), and WHR (*r* = 0.129, *p*=0.04), however, TC (*r* = −0.138, *p* = 0.03), LDL-C (*r* = −0.136, *p*=0.03) and eGFR (*r* = −0.022, *p* < 0.01) levels were significantly decreased as TNC levels increased. Serum alarin levels were significantly positively correlated with BMI (*r* = 0.253, *p* < 0.01), waist circumference (*r* = 0.241, *p* < 0.01) and hip circumference (*r* = 0.216, *p* < 0.01). The complete results of spearman correlation analysis are shown in Table S1.

Univariate logistic regression analysis revealed that TNC, alarin, age, waist circumference, WHR, HbAlc, TC, LDL-C, eGFR, duration of diabetes and hypertension were factors affecting CVD (*p* < 0.05). Multivariate logistic regression analysis confirmed that TNC (odds ratio (OR) 1.012; 95% confidence interval (CI) 1.000–1.023; *p*=0.045), alarin (OR 1.028; 95% CI 1.014–1.043; *p* < 0.001) and hypertension (OR 3.165; 95% CI 1.569–6.383; *p*=0.001) are independent risk factors for CVD (Table 3).

**Figure 3:**
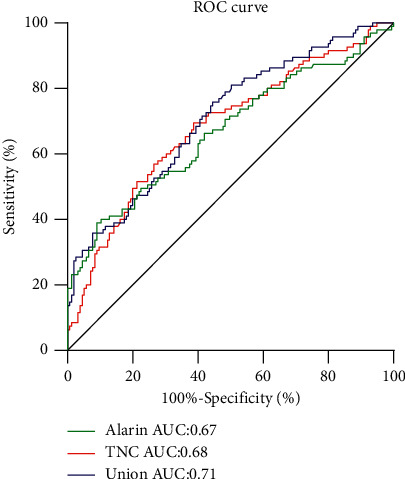
ROC curves for TNC and alarin in diabetics. The optimal cutoff point of TNC for CVD diagnosis in our study was >134.05 pg/mL (sensitivity 69.47%, specificity 61.29%). The best cutoff point of alarin was >142.69 pg/mL (sensitivity 38.95%, specificity 90.97%). AUC, area under the curve; CVD, cardiovascular diseases; ROC, receiver operating characteristic; TNC, tenascin-C.

ROC curve analysis showed that at a cutoff point of 134.05 pg/mL, TNC predicted a higher risk of CVD, with a sensitivity of 69.47% and a specificity of 61.29% (the area under the curve was 0.68) and the best cutoff of alarin was 142.69 pg/mL with a sensitivity of 38.95% and a specificity of 90.97% (the area under the curve was 0.67). Based on the regression equation, joint predictors were calculated from TNC and alarin. The discriminative ability of joint predictors (the area under the curve was 0.71) for determination of CVD in T2DM was higher than single TNC or single alarin (Figure 3).

When CVD individuals were divided into two categories, the first one including patients with one kind of CVD (*n* = 74) and the second including patients with at least two types of CVD (*n* = 21), TNC and alarin levels were significantly and independently associated with the extent of CVD in both crude and adjusted models (Table 4).

## 4. Discussion

We found that compared to the non-CVD group, serum TNC and alarin levels were significantly elevated in diabetic patients with CVD. After adjusting for various confounders, TNC and alarin levels were established to be independent determinants for the occurrence and severity of CVD in T2DM patients, suggesting that they contribute to the occurrence and development of diabetes-associated cardiovascular diseases. Moreover, TNC and alarin levels were respectively associated with many known cardiovascular disease risk factors.

As expected, elevated levels of chronic diseases were documented in the CVD group. There were no significant differences in BMI between the two groups, however, the CVD group exhibited a bigger waist circumference. This finding implies that abdominal obesity without generally being obese, may be associated with an elevated risk for CVD, consistent with a population-based longitudinal study in South Korea [23]. Interestingly, the CVD group exhibited improved lipid parameters and glycemic control, which was attributed to statins-use and insulin therapy after the occurrence of cardiovascular diseases.

Tenascin-C is a potential biomarker for predicting the occurrence and severity of coronary atherosclerosis as well as stroke severity and outcomes [24, 25]. However, in patients with dysglycemia, these conclusions are incompatible. TNC is associated with various chronic inflammatory diseases, including diabetes. Moreover, T2DM progression is enhanced by inflammation-induced ECM remodeling [6]. TNC overexpression is a critical point of ECM remodeling [26]. Therefore, TNC is involved in diabetes-associated complications and in its pathogenesis. Our results confirmed that TNC is a valuable biomarker for predicting the occurrence and severity of cardiovascular diseases in T2DM patients.

The precise role of TNC in cardiovascular disease development has not been clearly established. Atherosclerosis is a common feature in T2DM patients, and it results in severe cardiovascular diseases [27]. The potential mechanisms may be, first, during vascular injury, different cells, such as macrophages and smooth muscle cells, are the origins of tenascin-C [28, 29]. Second, tenascin-C expression changes smooth muscle cells from a non-proliferative phenotype to a migratory, synthetic state [30], and the degraded fragments of tenascin-C mediate smooth muscle cell apoptosis [31]. Third, a positive feed-forward loop between tenascin-C and matrix metalloproteases results in plaque progression, destabilization, and rupture [32]. These evidence indicate that during the pathological processes of atherosclerosis, tenascin-C expression is persistent, where its expression correlates with inflammation and plaque evolution. In addition, TNC upregulation after stroke contributes to activation of signaling transduction and exacerbation of secondary brain injuries through the toll-like receptor signaling pathway [33].

Alarin, a novel orexigenic peptide, was first identified in the gangliocytes of human neuroblastoma. The vasoactive, reproductive system, antibacterial, and antidepressant effects of alarin have been reported [15, 16, 34, 35]. A study from Turkey first confirmed the association of serum alarin levels with different ovarian reserve patterns. Elevated circulating alarin level, associating with increased LH concentration, was found in infertile women with poor ovarian reserve patterns [36]. Further research by this team confirmed a higher serum alarin concentration in the infertile women diagnosed with polycystic ovary syndrome (PCOS) and a positive correlation between serum alarin and LH level only in the PCOS women rather than other unexplained infertile [37]. To our knowledge, this is the first study to evaluate the relationship between serum alarin levels and the risk of CVD.

Alarin has been implicated in obesity and insulin sensitivity [19]. We found that alarin levels were significantly positively correlated with BMI, WC and HC. This finding is attributed to the failure of body to use insulin, as a result of decreased insulin sensitivity. Insulin resistance is an important pathological basis of diabetes mellitus, but also an important cause of CVD. Moreover, insulin resistance is critically associated with abnormalities in endothelial functions, which is the earliest finding in the pathogenesis of atherosclerosis [38]. Given the vital roles of endothelium-derived nitric oxide and the endothelium in maintenance of vascular tension, platelet adhesiveness and smooth muscle cell proliferation, endothelial dysfunction may contribute to increased risks of atherosclerosis in insulin-resistant individuals.

However, mechanisms of increased alarin levels in CVD patients remain elusive. After adjusting for age and BMI in patients without access to any lipid-lowering agents, Fang et al. reported that circulating alarin levels were significantly correlated with adverse lipid profiles [18]. We found that statins or fibrates may inhibit this association. Given that CVD groups present higher alarin levels as well as higher proportions of statin-users, we can infer that alarin and abnormal metabolism of serum lipids are interrelated. Besides, alarin was shown to increase adiponectin levels in type 2 diabetic rats [39]. Adiponectin can increase high-density lipoprotein levels and reduce very low-density lipoprotein as well as triglyceride levels in blood [40]. We postulated that elevated serum alarin levels might be a compensatory mechanism for metabolic stress. Therefore, when an individual has higher serum alarin levels, it is more likely to be as a result of abnormal lipid metabolism, which is a major pathophysiologic mechanism of atherosclerosis. Besides, adiponectin enhances inflammation under some chronic inflammatory conditions [41, 42]. Alarin is positively correlated with serum tumor necrosis factor-*α*, an inflammatory circulating molecule [18]. Therefore, we cannot rule out the possibility that alarin is involved in vascular diseases via enhancing inflammation in diabetic patients. Moreover, alarin could attenuate oxidative stress in heart failure rats [43]. High oxidative stress levels seem to be involved in the pathophysiology of various diseases, including diabetes, inflammatory diseases, cancers, and especially atherosclerosis [44, 45]. These results suggest that elevated alarin levels can be used to assess atherosclerosis progression.

### 4.1. Limitations of This Study

This study has several limitations. First, as a cross-sectional study, it is difficult to establish a causal relationship; therefore, cohort studies are needed. Meanwhile, cross-sectional detections do not represent stable levels of serum tenascin-C and alarin. Second, the enrolled participants were from single centers, and were hospitalized patients; therefore, our findings should be verified in other diabetic populations and in patients from different regions. Third, we did not have healthy controls.

## 5. Conclusions

In conclusion, circulating tenascin-C and alarin levels are significantly and independently associated with the occurrence and clinical severity of CVD in T2DM patients. These two biomarkers are potential diagnostic and prognostic indicators for CVD in diabetic patients. In addition, studies should evaluate the pathophysiological and molecular mechanisms of tenascin-C and alarin in atherosclerosis, which may inform on novel therapeutic directions to manage T2DM complications and morbidities.

## Figures and Tables

**Table 1:** Comparison of the clinical data between CVD group and non-CVD group.

Variable	CVD (*n* = 95)	Non-CVD (*n* = 155)	*p* value
Sex, M/F	58/37	105/50	0.281
Age, year	66 (56–73)	55 (45–66)	<0.001
BMI, kg·m^−2^	24.40 (22.66–27.00)	24.22 (22.10–26.45)	0.187
WC, cm	94 ± 10	90 ± 10	0.004
HC, cm	98 (94–104)	97 (94–101)	0.124
WHR	0.94 (0.90–0.98)	0.93 (0.89–0.96)	0.054
Smokers	30 (31.58%)	51 (32.90%)	0.828
Duration of diabetes, year	10.0 (5.0–20.0)	4.0 (0.3–10.0)	<0.001
HbA1c, %	9.6 (8.2–11.6)	10.5 (8.8–12.1)	0.022
FBG, mmol/L	7.52 (6.19–9.60)	7.47 (6.24–9.43)	0.806
FCP, ng/mL	1.28 (0.70–2.00)	0.99 (0.54–1.70)	0.074
2hCP, mIU/L	3.33 (2.21–5.47)	3.2 (2.19–4.84)	0.383
HOMA-IR	4.34 (2.37–6.53)	3.79 (2.01–6.19)	0.447
TG, mmol/L	1.33 (1.09–2.14)	1.47 (1.07–2.07)	0.396
TC, mmol/L	4.00 (3.48–5.17)	4.82 (4.17–5.60)	<0.001
LDL-C, mmol/L	2.34 ± 0.96	2.97 ± 0.92	<0.001
HDL-C, mmol/L	0.93 (0.77–1.19)	0.96 (0.84–1.13)	0.342
UA, *μ*mol/L	334.8 (267.5–428.1)	333.7 (271.8–403.8)	0.805
eGFR, ml/min/1.73 m^2^	97 (83–109)	109 (92–117)	<0.001
DR	28 (29.47%)	31 (20.00%)	0.087
DN	17 (17.89%)	8 (5.16%)	0.001
Hypertension	69 (72.63%)	57 (36.77%)	<0.001
TNC, pg/mL	144.51 (122.99–182.47)	127.24 (108.07–142.65)	<0.001
Alarin, pg/mL	126.59 (105.95–152.63)	107.90 (98.37–126.21)	<0.001

CVD, cardiovascular diseases; M/F, male/female; BMI, body mass index; WC, waist circumference; HC, hip circumference; WHR, waist-hip ratio; HbA1c, glycated hemoglobin; FBG, fasting blood glucose; FCP, fasting C-peptide; 2hCP, 2 h postprandial C-peptide; HOMA-IR, homeostasis model assessment of insulin resistance index; TG, triglycerides; TC, total cholesterol; LDL-C, low-density lipoprotein cholesterol; HDL-C, high-density lipoprotein cholesterol; UA, uric acid; eGFR, estimated glomerular filtration rate; DR, diabetic retinopathy; DN, diabetic nephropathy; TNC, tenascin-C.

**Table 2:** Comparison of the medications between CVD groups and non-CVD group.

Medications	CVD	Non-CVD	*p* value
Antihypertension drugs	(*n* = 69)	(*n* = 57)	
*β*-blockers	33.33	21.00	0.126
Diuretics	30.43	10.53	0.007
Calcium-channel blockers	49.28	50.88	0.858
RAAS inhibitors	68.12	43.86	0.006
Others	7.25	8.77	0.753
Lipid-lowering drugs	(*n* = 67)	(*n* = 59)	
Statins	98.51	89.83	0.034
Fibrates	1.49	6.78	0.129
Antidiabetic drugs	(*n* = 95)	(*n* = 155)	
Diet	2.11	1.29	0.618
Oral drugs	76.84	67.74	0.123
Insulin	49.47	28.39	0.001

Data are percentage unless otherwise indicated. CVD, cardiovascular diseases; RAAS, renin–angiotensin–aldosterone system.

**Table 3:** Multivariate logistic regression analysis of factors affecting CVD.

Variable	*β*	SE	Wald	*p*	OR	95% CI
Age	0.026	0.016	2.851	0.091	1.027	0.978–1.059
WC	0.000	0.020	0.000	0.989	1.000	0.962–1.040
WHR	3.422	2.883	1.409	0.235	30.638	0.108–8712.843
Duration of diabetes	0.030	0.022	1.841	0.175	1.031	0.987–1.077
HbAlc	−0.030	0.062	0.231	0.631	0.971	0.859–1.096
TC	−0.046	0.243	0.036	0.850	0.955	0.593–1.537
LDL-C	−0.513	0.300	2.937	0.087	0.598	0.333–1.077
eGFR	0.007	0.009	0.604	0.437	1.007	0.990–1.024
Hypertension	1.152	0.358	10.358	0.001	3.165	1.569–6.383
TNC	0.012	0.006	4.018	0.045	1.012	1.000–1.023
Alarin	0.028	0.007	15.576	<0.001	1.028	1.014–1.043

CI, confidence interval; CVD, cardiovascular diseases; eGFR, estimated glomerular filtration rate; HbA1c, glycated hemoglobin; LDL-C, low-density lipoprotein cholesterol; OR, odds ratio; SE, standard error; TC, total cholesterol; TNC, tenascin-C; WC, waist circumference; WHR, waist-hip ratio.

**Table 4:** Multivariable logistic regression models for severity of CVD.

Models	*p*	OR	95% CI
Tenascin-C			
Crude	0.006	1.023	1.004–1.040
Model 1	0.046	1.019	1.000–1.038
Model 2	0.018	1.028	1.005–1.052
Model 3	0.019	1.038	1.006–1.071
Alarin			
Crude	0.019	1.023	1.006–1.040
Model 1	0.007	1.032	1.000–1.056
Model 2	0.003	1.046	1.015–1.077
Model 3	0.003	1.053	1.018–1.089

Model 1, adjusted for age, sex, BMI and WHR; Model 2, adjusted for Model 1+ duration of diabetes, HbAlc, HOMA-IR, DN and DR; Model 3, adjusted for Model 2+ TG, TC, LDL-C, HDL-C, hypertension and smoking status. BMI, body mass index; CI, confidence interval; CVD, cardiovascular diseases; DN, diabetic nephropathy; DR, diabetic retinopathy; HbA1c, glycated hemoglobin; HDL-C, high-density lipoprotein cholesterol; HOMA-IR, homeostasis model assessment of insulin resistance index; LDL-C, low-density lipoprotein cholesterol; OR, odds ratio; TC, total cholesterol; TG, triglycerides; WHR, waist-hip ratio.

## Data Availability

The data used to support the findings of this study are available from the corresponding author upon request.

## Supplementary Materials

Supplementary Materials Table S1: Spearman correlation analysis of serum tenascin-C and alarin level with clinical data.
