# Supplementary material for: Serum Tenascin-C and Alarin Levels Are Associated with Cardiovascular Diseases in Type 2 Diabetes Mellitus
Source: Int J Endocrinol. 2022 Apr 21;2022:2009724. doi: 10.1155/2022/2009724 (PMC9050282; doi:10.1155/2022/2009724)
Supplement: Supplementary Materials — Table S1: Spearman correlation analysis of serum tenascin-C and alarin level with clinical data. [file 2009724.f1.docx]

Table S1: Spearman correlation analysis of serum tenascin-C and alarin level with clinical data.

| Parameter | Tenascin-C | | Alarin | |
| --- | --- | --- | --- | --- |
|  | r | *p* | r | *p* |
| Age | 0.241 | <0.001 | 0.043 | 0.497 |
| BMI | 0.108 | 0.088 | 0.253 | <0.001 |
| WC | 0.142 | 0.024 | 0.241 | 0.001 |
| HC | 0.104 | 0.100 | 0.216 | 0.001 |
| WHR | 0.129 | 0.041 | 0.105 | 0.097 |
| Duration of diabetes | 0.065 | 0.309 | 0.011 | 0.865 |
| HbAlc | −0.011 | 0.078 | −0.033 | 0.606 |
| FBG | 0.113 | 0.074 | 0.045 | 0.481 |
| FCP | 0.123 | 0.054 | 0.119 | 0.060 |
| 2hCP | 0.094 | 0.140 | 0.043 | 0.501 |
| HOMA-IR | 0.096 | 0.128 | 0.105 | 0.098 |
| TG | −0.038 | 0.545 | 0.023 | 0.717 |
| TC | −0.138 | 0.029 | −0.033 | 0.602 |
| HDL-C | −0.080 | 0.210 | 0.087 | 0.170 |
| LDL-C | −0.136 | 0.032 | −0.047 | 0.464 |
| UA | 0.017 | 0.783 | 0.033 | 0.605 |
| eGFR | −0.022 | <0.001 | −0.080 | 0.207 |

2hCP, 2 h postprandial C-peptide; BMI, body mass index; eGFR, estimated glomerular filtration rate; FBG, fasting blood glucose; FCP, fasting C-peptide; HbA1c, glycated hemoglobin; HC, hip circumference; HDL-C, high-density lipoprotein cholesterol; HOMA-IR, homeostasis model assessment of insulin resistance index; LDL-C, low-density lipoprotein cholesterol; TC, total cholesterol; TG, triglycerides; UA, uric acid; WC, waist circumference; WHR, waist-hip ratio.
